# Supplementary material for: RNAi-mediated knockdown of two orphan G protein-coupled receptors reduces fecundity in the yellow fever mosquito Aedes aegypti
Source: Front Insect Sci. 2023 Aug 23;3:1197945. doi: 10.3389/finsc.2023.1197945 (PMC10926455; doi:10.3389/finsc.2023.1197945)
Supplement: Supplementary file 1 [file DataSheet_1.zip › Figure S1.PDF]

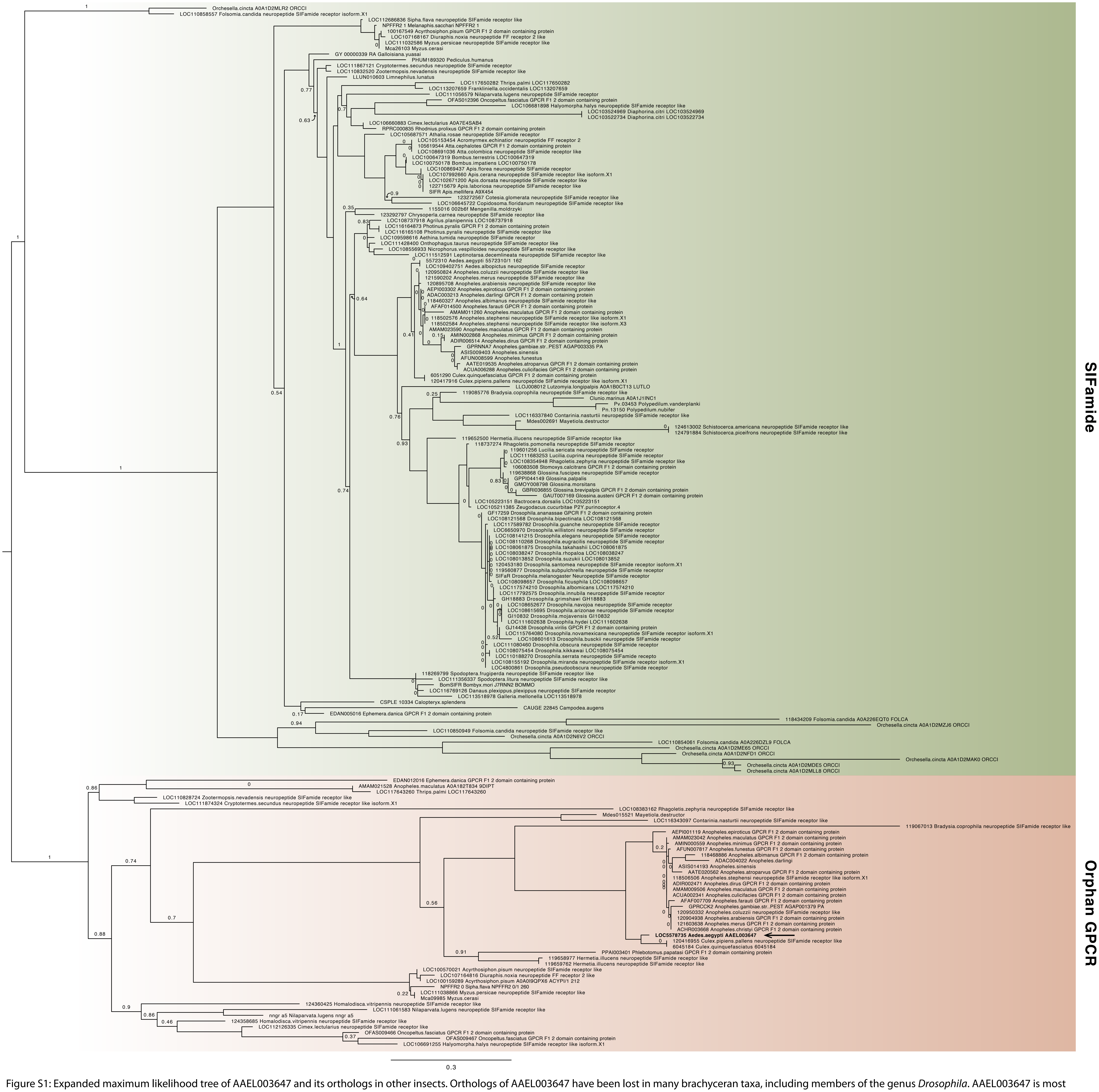

Figure S1: Expanded maximum likelihood tree of AAEL003647 and its orthologs in other insects. Orthologs of AAEL003647 have been lost in many brachyceran taxa, including members of the genus *Drosophila*. AAEL003647 is most closely related to the SIFamide receptor. Sequences were downloaded from OrthoDB and aligned against a 7 transmembrane GPCR model (7tm-1.hmm) in hmmalign. Trees were built in PhyML. Support values are aLRT SH-like, and branches with support values < 0.95 are labeled with their support values. F1 2 domain containing protein is abbreviated as "F1 2 dom. con't. prot."
