# Supplementary material for: RNAi-mediated knockdown of two orphan G protein-coupled receptors reduces fecundity in the yellow fever mosquito Aedes aegypti
Source: Front Insect Sci. 2023 Aug 23;3:1197945. doi: 10.3389/finsc.2023.1197945 (PMC10926455; doi:10.3389/finsc.2023.1197945)
Supplement: Supplementary file 1 [file DataSheet_1.zip › Figure S2.pdf]

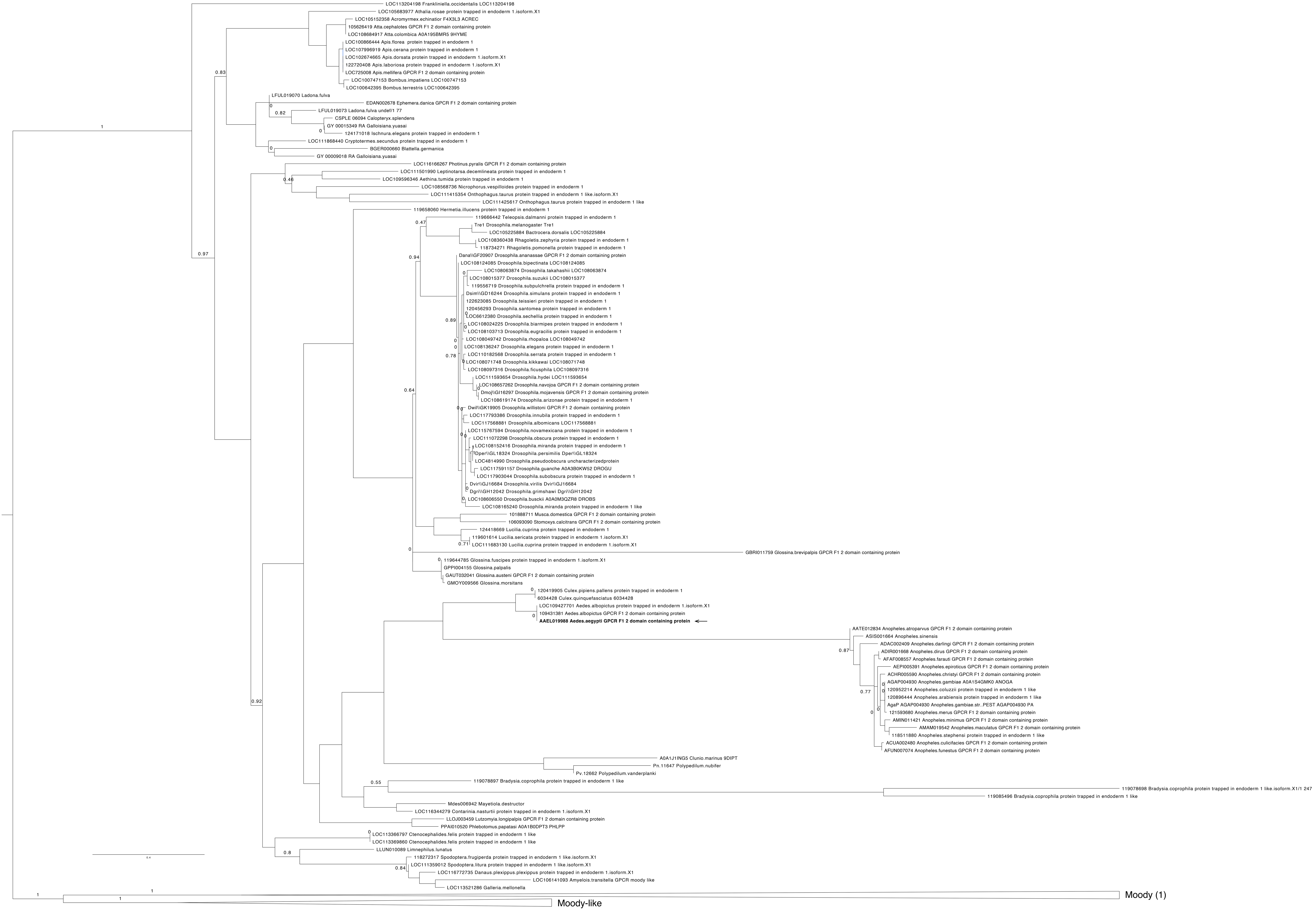

Figure S2: Expanded maximum likelihood tree of AAEL019988 and its orthologs in other insects. Genome sequences of most hemimetabolous insects do not encode an ortholog of AAEL019988. Sequences were downloaded from OrthoDB and aligned against a 7 transmembrane GPCR model (7tm-1.hmm) in hmmalign. Trees were built in PhyML. Support values are aLRT SH-like, and branches with support values < 0.95 are labeled with their support values.
